# Ubiquitination in Scleroderma Fibrosis and Its Treatment

**DOI:** 10.3389/fimmu.2018.02383

**Published:** 2018-10-17

**Authors:** Ying Long, Weilin Chen, Qian Du, Xiaoxia Zuo, Honglin Zhu

**Affiliations:** Department of Rheumatology, Xiangya Hospital, Central South University, Changsha, China

**Keywords:** SSc, ubiquitination, TGF-β, WNT/β-catenin, STAT3

## Abstract

Scleroderma (systemic sclerosis, SSc) is a highly heterogeneous rheumatic disease, and uncontrolled fibrosis in visceral organs is the major cause of death in patients. The transforming growth factor-β (TGF-β) and WNT/β-catenin signaling pathways, along with signal transducer and activator of transcription 3 (STAT3), play crucial roles in this fibrotic process. Currently, no therapy is available that effectively arrests or reverses the progression of fibrosis in patients with SSc. Ubiquitination is an important post-translational modification that controls many critical cellular functions. Dysregulated ubiquitination events have been observed in patients with systemic lupus erythematosus, rheumatoid arthritis and fibrotic diseases. Inhibitors targeting the ubiquitination pathway have considerable potential for the treatment of rheumatic diseases. However, very few studies have examined the role and mechanism of ubiquitination in patients with SSc. In this review, we will summarize the molecular mechanisms of ubiquitination in patients with SSc and explore the potential targets for treatment.

## Introduction

Scleroderma (systemic sclerosis, SSc) is a complicated heterogeneous rheumatic disease that is characterized by progressive fibrosis in the skin and multiple other organs. Both environmental and genetic factors contribute to the etiology of SSc and trigger a chronic self-amplifying inflammatory process, leading to vascular alterations, autoimmunity and fibrosis (1). Many molecules and signaling pathways participate in the progression of fibrosis, such as the transforming growth factor-β (TGF-β) and WNT/β-catenin signaling pathways, signal transducer and activator of transcription 3 (STAT3), platelet-derived growth factor (PDGF), endothelin 1, interleukin 6, interleukin 13, autoantibodies, and numerous biologically active substances. Among these pathways, the TGF-β and WNT/β-catenin signaling pathways and STAT3 play key roles. Based on accumulating evidence, post-translational modifications have important regulatory roles in these pathways, such as acetylation, phosphorylation and ubiquitination, suggesting that these modifications are potential targets for the treatment of fibrosis (2–4).

Ubiquitin is a highly evolutionarily conserved protein that modifies other proteins for degradation. Ubiquitination is a process by which target protein is covalently bound to ubiquitin through an enzymatic cascade that is orchestrated sequentially by activating (E1), conjugating (E2) and ligating (E3) enzymes (5). E1 enzymes activate ubiquitin and transfer it onto the E2 conjugating enzyme, and then E3 ligases interact with the ubiquitin-loaded E2 enzyme and substrate protein to mediate the formation of polyubiquitin chains. Subsequently, the polyubiquitin chain is recognized by the 26S proteasome complex and degraded into individual amino acids.

The human genome contains two E1 enzymes, approximately forty E2 enzymes and >600 E3 ligases. The diverse E3 ligases have important roles in the selective recognition of each targeted protein. Additionally, >100 deubiquitinating enzymes (DUBs) have been identified that remove ubiquitin from the substrate proteins (6).

Ubiquitination plays important roles in the proteasomal degradation of proteins, inflammatory signaling, immune responses, autophagy, and T cell activation and differentiation (7). Dysregulation of ubiquitination has been observed in many autoimmune diseases, such as systemic lupus erythematous (8), rheumatoid arthritis (RA) (9) and fibrotic diseases (10). Anti-ubiquitin antibodies are present in 42% of patients with SSc and are associated with anti-histone antibodies. The latter might be positively correlated with the severity of pulmonary fibrosis in patients with SSc (11). According to results from whole-exome sequencing, an E3 ubiquitin ligase-related gene was associated with a higher risk of SSc. Therefore, ubiquitination might play an important role in SSc (12). However, little is known about ubiquitination in the pathology of SSc. Herein, we will summarize the role of ubiquitination in SSc and then discuss the future perspectives for SSc therapy.

## Mutations in ubiquitination-related enzymes in patients with SSc

Many susceptibility regions have been identified in patients with SSc by genome-wide association studies (GWAS), and the most common and confirmed susceptibility locus is the HLA locus. Recently, non-HLA susceptibility genes have also been identified, and most are correlated with inflammation, T cell differentiation and autoantibodies (13).

The susceptibility genes TNF-α-induced protein 3 (TNFAIP3), TNF receptor-interacting protein (TNIP1), ankyrin repeat and SOCS box-containing 10 (ASB10), and autophagy-related 5 (ATG5) are involved in the ubiquitination-proteasome system (UPS). TNFAIP3 expression (encodes A20) is rapidly induced by TNF-α. TNFAIP3 possesses both E3 ubiquitin ligase and deubiquitinase activities and negatively regulates the inflammatory response by deubiquitinating proteins in the NF-κB pathway, such as IKKg/NEMO, RIP1 and RIP2. TNFAIP3 is associated with diffuse cutaneous SSc, anti-topoisomerase I antibody, lung fibrosis and pulmonary arterial hypertension (14). TNIP1 interacts with A20 and represses the activity of the TLR-induced NF-κB signaling pathway, decreasing the production of proinflammatory cytokines in patients with SSc. Recombinant TNIP1 downregulates inflammatory cytokine-induced collagen synthesis (15). ASB10 belongs to the E3 ubiquitin ligase complex and may be involved in the pathogenesis of SSc and pulmonary vascular complications (12, 16). The protein encoded by the ATG5 gene interacts with ATG12 and forms a complex that functions as an E1-like activating enzyme. ATG5 is also associated with RA, juvenile idiopathic arthritis and primary biliary cirrhosis. However, the function of ATG5 in patients with SSc requires further investigation (17).

## Ubiquitin modification of proteins in key signaling pathways involved in SSc

### TGF-β signaling is regulated by ubiquitination

#### Definition

**Figure 1:**
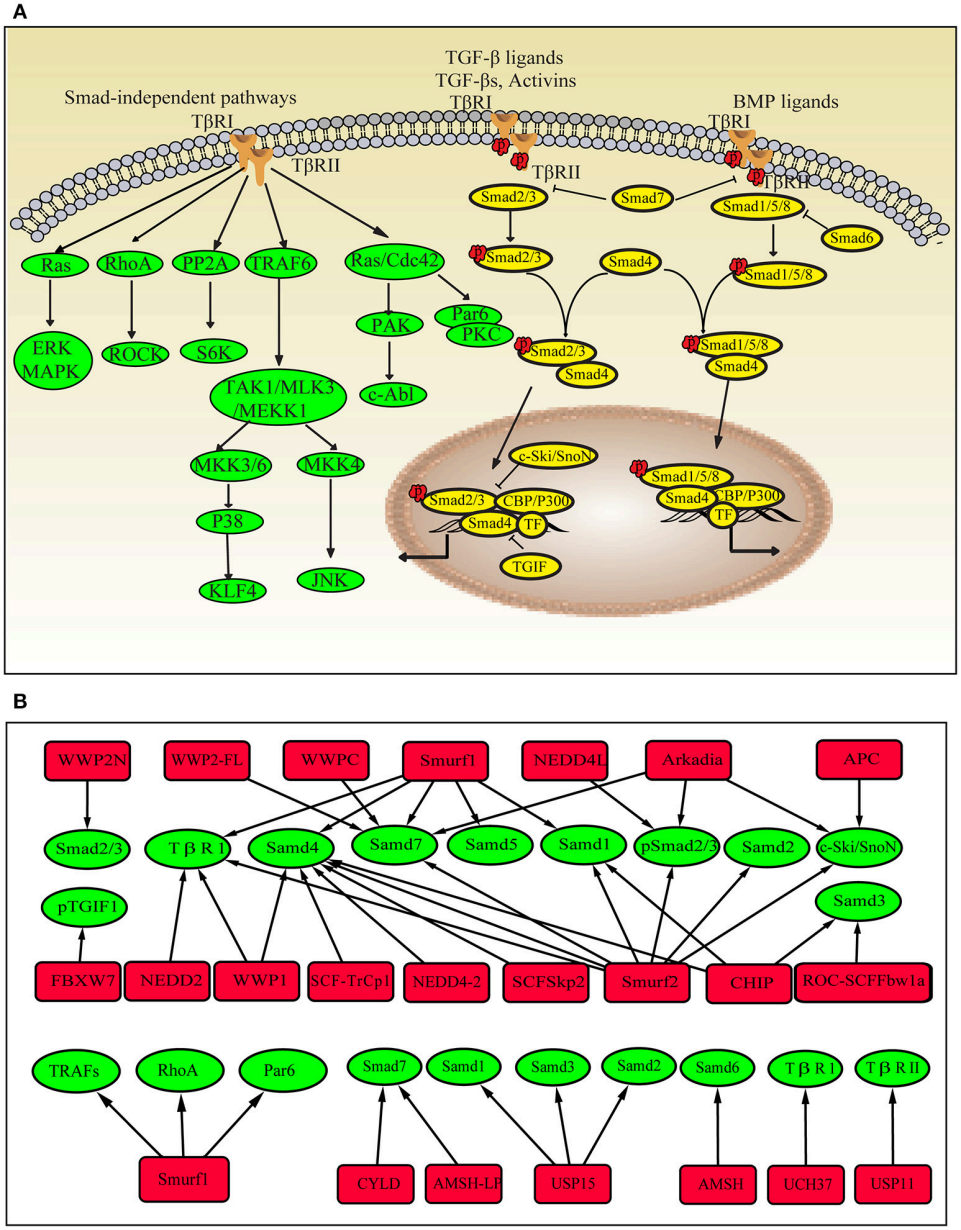
Ubiquitin modifications in the TGF-β pathway. **(A)** Schematic showing the Smad-dependent and Smad-independent TGF-β signaling pathways. **(B)** Ubiquitinating enzymes, DUBs and target proteins in the TGF-β pathway. Red square nodes represent enzymes, green circles represent target proteins.

The TGF-β superfamily consists of TGF-βs, bone morphogenetic proteins (BMPs) and activin. These proteins activate TGF-β signaling by binding to their membrane-anchored serine/threonine kinase receptors TGF-βRI or TGF-βRII. The canonical TGF-β signaling pathway is regulated by Smad proteins. Smad proteins are divided into three types. R-Smads are receptor-regulated Smads, including TGF-β/activin-specific Samd2 and Smad3, BMP-specific Smad1, Smad5 and Smad8. I-Smads are inhibitory Smads and include Smad6 and Smad7. Co-Smad is the common Smad and is represented by Smad4. The Smad proteins regulate the transcription of various genes. Many transcription factors and co-factors are also involved in this process; transcription factors include Mixer, FoxH1, E2F, and Runx-related proteins, co-activators include p300 and CBP, and co-repressors include c-Ski and SnoN (18) (Figure 1A).

#### The role of TGF-β signaling in SSc

TGF-β is commonly viewed as playing a critical role in the fibrosis process. Dysregulation of the TGF-β signaling pathway is involved in the pathogenesis of SSc (19). High levels of TGF-β and its regulated genes have been detected in skin biopsies and were positively correlated with the severity of SSc (20). The TGF-β neutralizing antibody fresolimumab exerts anti-fibrotic effects on patients with SSc, but its use is also accompanied by a high incidence of keratoacanthomas, which limits its use in long-term treatment. Therefore, the development of new drugs that target downstream mediators of TGF-β signaling is important (21, 22).

#### Ubiquitin enzymes in TGF-β/SMAD signaling

Many E3 ligases are involved in TGF-β/SMAD signaling, including Smurfs (E6-accessory protein C-terminus, HECT), WWP family (HECT type), NEDD4L (HECT type), Arkadia (RING-H2 finger domain), CHIP (C-terminus of HSC70-interacting protein), β-TrCP (Skp1-Cullin-F-box (SCF)-type ubiquitin ligase), and Fbxw7 (SCF type). TGF-β/SMAD signaling regulates the transcription of various genes, including negative regulators, such as I-Smads and Smurfs. When TGF-β signaling is activated, I-Smads and Smurfs interact in the nucleus and translocate to the cytoplasm (23, 24). Smad7 recruits WWP1 and NEDD4L to the active TGF-β receptor complexes and induces the degradation of the complexes (25, 26). Smurf1 ubiquitinates Smad1 and Smad5 (27, 28), and Smurf2 ubiquitinates Smad1 and Smad2 under steady-state conditions (29, 30). Arkadia and NEDD4L ubiquitinate phospho-Smad2/3 (31, 32), whereas CHIP regulates the abundance of Smad1 and Smad3 (33, 34). Smurfs, WWP1, NEDD4-2, CHIP, and β-TrCP conjugate polyubiquitin chains onto Smad4 and mediate its degradation (35). As mentioned above, E3 ligases mainly function as inhibitors of TGF-β signaling. However, they have also been shown to enhance TGF-β signaling. Arkadia degrades the negative regulators of TGF-β signaling, such as Smad7, c-Ski, and SnoN (36). Smad7 is also ubiquitinated by WWP-C and WWP2-FL (37). Phosphorylated TGIF1 (TGF-β-induced factor 1) is a transcriptional repressor of TGF-β signaling that is degraded by the ubiquitin ligase complex containing Fbxw7 (38).

#### Ubiquitin enzymes in Smad-independent TGF-β signaling

Ubiquitination also plays important roles in Smad-independent TGF-β pathways. TGF-β induces the ubiquitination and degradation of KLF4 (Krüppel-like factor 4), which is important for TGF-β-mediated regulation of transcription (39). The TGF-β/RhoA pathway is required for the progression of the epithelial-mesenchymal transition (EMT). Smurf1 targets RhoA for degradation (40). In TGF-β-induced anti-inflammatory signaling, Smurf1 ubiquitinates TRAFs (TNF receptor-associated factors), and Smurf2 interacts with TRAF2 and ubiquitinates TNF receptor 2, thereby inhibiting downstream signaling (41). In fibroblasts from patients with SSc, Smurf2 is upregulated after stimulation with TGF-β (42), and the Smad7-Smurf-mediated inhibitory effect is impaired (43). Ubiquitination also promotes Smad2/3 signaling and increases collagen I accumulation by stabilizing Ha-Ras, which is independent of TGF-β activation. All of these mechanisms eventually contribute to collagen overproduction. The dysregulation of E3 ubiquitin ligases involved in TGF-β signaling has also been observed in patients with other fibrotic diseases and in animal models. The levels of MDM2 (RING-type) and FIEL1 (HECT-Type E3) are increased in lung tissues from patients with idiopathic pulmonary fibrosis (IPF) (44, 45). Smurf2 is upregulated in the fibroblasts present in hypertrophic scars (46). Smurf1, Smurf2, Arkadia and Hrd1 (Synoviolin, RING-type) levels are increased in animals with unilateral ureteral obstruction-induced renal fibrosis (47–49). Smurf2 and Synoviolin are upregulated in a liver fibrosis model (50–52). NEDD4 and Pellino1 (RING-type) expression are increased in keloid fibroblasts (53) and cardiac fibroblasts, respectively (54). These E3 ubiquitin ligases not only directly mediate the degradation of components of the TGF-β signaling pathway but also participate in inducing the transition of epithelial cells to mesenchymal cells, enhancing fibroblast proliferation and invasiveness, and increasing TGF-β production.

#### DUBs

DUBs have also been implicated in TGF-β signaling. UCH37 and USP11 were shown to deubiquitinate TβRI or TβRII, which is important for early steps in the TGF-β signaling pathway (55–57). High levels of USP11 have been detected in lung tissues from patients with IPF and bleomycin-induced mice, whereas inhibition of USP11 expression attenuates TGF-β signaling (57). CYLD deubiquitinates Smad7 and inhibits TGF-β signaling (58). In mice with liver fibrosis, CYLD ameliorates hepatocellular damage and liver fibrogenesis (59). USP15 deubiquitinates mono-ubiquitinated R-Smads and is required for proper TGF-β signaling. Other DUBs, such as AMSH (the associated molecule with the Src homology 3 domain of the signal-transducing adaptor molecule) and AMSH-like protein cleave K63-linked ubiquitin chains, which are associated with I-Smads and inhibit their functions (60–62) (Figure 1B).

### The WNT/β-catenin pathway is regulated by ubiquitination

#### Definition

The canonical WNT pathway is closely related to the regulation of β-catenin and its potential to modulate transcription. When WNT signaling is inactivated, β-catenin is phosphorylated (pβ-catenin) in the cytoplasm by a multiprotein complex (Axin/APC complex) composed of Axin, APC, CK1 and GSKβ. The pβ-catenin protein is immediately degraded by the UPS and is rarely detected in normal cells. Upon stimulation, WNT binds to the receptor Frizzled (Fz) and the coreceptors LRP5/6, recruits the cytoplasmic effector protein Disheveled (Dvl) and inhibits the Axin/APC complex. Consequently, the high levels of cytosolic β-catenin are translocated to the nucleus and regulate the transcription of target genes via β-catenin/TCF complexes. Based on accumulating evidence from recent studies, ubiquitin modification also plays important roles in regulating the WNT pathway (63).

#### The role of WNT/β-catenin pathway in SSc

WNT signaling plays pivotal roles in developmental processes and tissue homeostasis. The canonical WNT/β-catenin pathway elicits fibrotic responses both directly and through TGF-β (2). High levels of activated β-catenin and its regulated gene AXIN2 have been in skin and lung tissues from patients with SSc, as well as in animal models of fibrosis (64, 65). SSc autoantibodies and oxidative DNA damage mediate Wnt inhibitor factor 1 (WIF-1) silencing and promote WNT activation and subsequent fibrosis. Strategies that restore the expression of WIF-1 prevent collagen accumulation *in vivo*. Microarray studies have also revealed the activation of WNT/β-catenin pathways in the skin tissues from patients with SSc. According to the results from clinical trials, treatments targeting the WNT/β-catenin pathway (tankyrase and porcupine inhibitors) are effective, well-tolerated and safe for long-term application (66). C-82, which targets the β-catenin/CBP interaction, is now in phase I/II clinical trials for SSc therapy. Therefore, the molecular mechanisms regulating the WNT/β-catenin pathway must be completely identified (67, 68).

#### Ubiquitin enzymes and DUBs in WNT signaling

**Figure 2:**
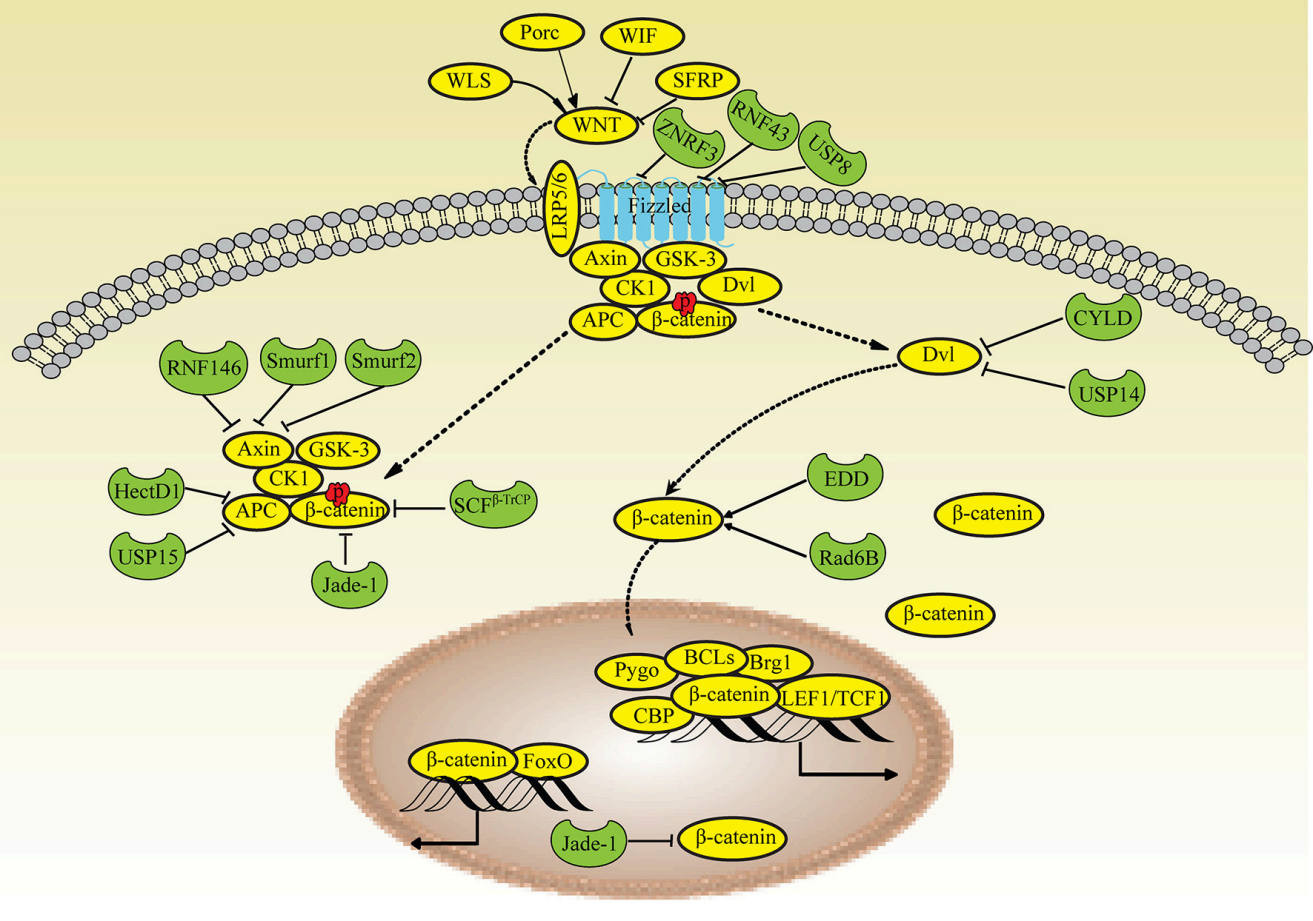
Ubiquitin modifications in the WNT/β-catenin pathway. When WNT signaling is inactivated, β-catenin is phosphorylated (pβ-catenin) in the cytoplasm by the Axin/APC complex and degraded by Jade-1 and β-TrCP. Upon stimulation, WNT binds to the receptor Frizzled and the coreceptors LRP5/6, recruits Dvl and inhibits the Axin/APC complex, leading to the translocation of high levels of cytosolic β-catenin into the nucleus. Fz is ubiquitinated by ZNRF3 and RNF43 and deubiquitinated by USP8. Dvl is deubiquitinated by CYLD and USP14. Axin is degraded by RNF146, Smurf2, and Smurf1. APC is ubiquitinated by HectD1 and deubiquitinated by USP15. Rad6B and EDD ubiquitinate β-catenin; Jade-1 also ubiquitinates β-catenin in the nucleus.

The cell-surface receptor Fz is ubiquitinated by the transmembrane E3 ligases ZNRF3 and RNF43 and is deubiquitinated by UBPY/Ub-specific protease 8 (USP8) for recycling to the plasma membrane (69, 70). LRP6 is retained in the endoplasmic reticulum due to palmitoylation and monoubiquitylation, suggesting that an E3 ligase and DUBs participate in this process; however, the types of ubiquitin chains remain unknown (71). Dvl proteins (Dvl1, Dvl2 and Dvl3) play key roles in both canonical and noncanonical WNT signaling. Multiple E3 ligases that ubiquitinate Dvl negatively regulate WNT signaling (72). The DUBs CYLD and USP14 remove the K63-linked polyubiquitin chain from Dvl (73, 74). Axin is degraded by the E3 ligase RNF146 and Smurf1 and Smurf2, which interact with LRP5/6 (75–77). HectD1 ubiquitinates APC, promotes its interaction with Axin and negatively regulates WNT signaling (78), whereas USP15 protects the APC from ubiquitin-mediated degradation (79). β-TrCP assembles K48-linked polyubiquitin chains onto β-catenin and mainly regulates the nuclear pool of β-catenin (80). The ubiquitin ligase Jade-1 also mediates β-catenin ubiquitination and is responsible for degrading cytoplasmic β-catenin (81). Unlike β-TrCP and Jade-1, Rad6B (an E2 ubiquitin-conjugating enzyme) and EDD (an E3 ubiquitin ligase) ubiquitinate β-catenin and increase its activity (82, 83) (Figure 2).

### STAT3 regulation by ubiquitination

STAT3 belongs to the transcription factor family that transduces cellular signals from a number of soluble growth factors and cytokines, including PDGF, epidermal growth factor (EGF) and IL-6 family cytokines. STAT3 plays critical roles in several biological processes, including cell proliferation, differentiation and migration. Upon stimulation, cytoplasmic STAT3 is phosphorylated, dimerizes, and then translocates to the nucleus to regulate the transcription of target genes. Recently, STAT3 was shown to integrate multiple profibrotic signals and was identified as a key checkpoint in fibroblast activation. STAT3 is considered a potential target for SSc treatment (4, 84). The STAT3 dimerization inhibitor S3I-201 exerts strong anti-fibrotic effects on animal models of SSc. STAT3 is ubiquitinated with lysine-63-linked ubiquitin chains by TRAF6 (tumor necrosis factor receptor-associated factor 6) (85), which exhibits E3 ubiquitin ligase activity. STAT3 is also ubiquitinated and degraded by the E3 ligase COP1 (86).

### Treatment of SSc by strategies targeting the UPS

Bortezomib is a reversible 20S proteasome inhibitor and the first drug approved to treat multiple myeloma. In the UPS, the proteasome is the final step in protein degradation and a valuable target for developing potential drugs. Bortezomib derivatives, such as carfilzomib and marizomib, are in various phases of clinical trials as potential treatments for several malignancies (87). In fibroblasts from patients with SSc, proteasome inhibitors replenish human dermal fibroblasts, degrade the extracellular matrix and exert anti-fibrotic effects (88). The proteasome inhibitor MG-132, synthetic lactacystin, and bortezomib decrease the expression of type I collagen and tissue inhibitor of metalloproteinase-1 and increase the production of metalloproteinase-1 in a dose-dependent manner (89). However, proteasome inhibitors have been reported to induce resistance and side effects, and a combination of several UPS inhibitors targeting different components may overcome this challenge (6).

Studies of E1 enzyme inhibitors are rare due to their lack of specificity as therapies. Several inhibitors of E2 enzymes are in preclinical research stages, but their role in SSc has been poorly investigated. The specificity of the UPS depends mainly on E3 ubiquitin ligases. The FIEL1 inhibitor BC-1485 ameliorates lung fibrosis in a mouse model (44). The Synoviolin inhibitor LS-102 reduces endoplasmic reticulum stress-induced collagen secretion from lung epithelial cells, suggesting that it might be a potential treatment for IPF (90). In patients with SSc, the TGF-β and WNT/β-catenin signaling pathways and STAT3 are mainly regulated by E3 ligases at multiple levels, which presents the potential for specific substrates for drug target design. Erioflorin, which is isolated from *Eriophyllum lanatum*, has been shown to block β-TrCP and affect WNT/β-catenin signaling (91). Specific inhibitors or antagonists of other E3 ligases, such as Smurf1, Smurf2 and NEDD4, have not yet been discovered.

DUBs deubiquitinate and rescue substrates from proteasomal degradation, and thus are regarded other potential targets for drug development. WP1130 has been shown to suppress the activity of several DUBs, including UCH37 and USP14, which regulate both TGF-β and WNT/β-catenin signaling. The combination of WP1130 and bortezomib exerts pro-apoptosis and anti-proliferation effects on tumor cells (92). b-AP15 is a small-molecule inhibitor of USP14 and UCHL5. b-AP15 blocks USP14 in a reversible manner and regulates WNT/β-catenin signaling (93). UCHL5 levels are elevated in lung tissues from patients with IPF (80). b-AP15 also reduces the levels of the fibronectin, type I collagen, and SMAD2/3 proteins in lung tissues from mice with fibrosis (94). The USP11 inhibitor mitoxantrone attenuates TGF-β signaling in lung fibroblasts and has been indicated as a potential antifibrotic drug for subjects with fibrosis.

In animal models and clinical trials, the UPS has been validated as a valuable molecular target for the treatment of cancer, asthma and arthritis, as >1,000 proteins have been identified in the UPS. These proteins represent substantial opportunities and challenges for researchers to investigate the molecular mechanisms underlying the addition of ubiquitin chains and the main components. The identification and validation of these components will expand the pool of targets for drug discovery for fibrosis (95).

## Conclusions and future perspectives

In this review, we highlight the mechanisms regulating ubiquitination in patients with SSc and explore potential anti-fibrosis drugs. Effective therapies for many fibrotic manifestations in patients with SSc are currently unavailable. Considering the central role of TGF-β signaling, WNT/β-catenin signaling and STAT3 in SSc, the use of UPS inhibitors to selectively disrupt the formation of receptor or co-receptor complexes or block intracellular signaling may yield advances in the development of urgently needed treatments. These drugs are very powerful and might also induce severe side effects because of their unselective action that would limit their widespread use. In the near future, the elucidation of new, potent and highly specific drugs targeting specific UPS components is required. Therefore, investigations of the enzymology of ubiquitination will be of paramount importance in the next few years. Moreover, more studies are needed of enzymes involved in ubiquitination that represent promising drug targets to ameliorate fibrosis in patients with SSc.

## Author contributions

YL wrote the first draft. WC, QD, and XZ revised the manuscript. HZ revised the final version and inserted additional information.

### Conflict of interest statement

The authors declare that the research was conducted in the absence of any commercial or financial relationships that could be construed as a potential conflict of interest.
